# Afforested farmland vs. forestland: Effects of bark stripping by *Cervus elaphus* and climate on production potential and structure of *Picea abies* forests

**DOI:** 10.1371/journal.pone.0221082

**Published:** 2019-08-15

**Authors:** Jan Cukor, Zdeněk Vacek, Rostislav Linda, Ram Prasad Sharma, Stanislav Vacek

**Affiliations:** 1 Faculty of Forestry and Wood Sciences, Czech University of Life Sciences Prague, Kamýcká, Prague –Suchdol, Czech Republic; 2 Forestry and Game Management Research Institute, Strnady, Jíloviště, Czech Republic; Assam University, INDIA

## Abstract

The aim of this study was to evaluate (1) effects of bark stripping and climatic factors on radial growth of *Picea abies* /L./ Karst., (2) production and structural differences between stands established on the forestland and abandoned farmland (afforested farmland–henceforth, farmland), and (3) interaction among the losses caused by ungulate damages, production, diversity, and soil types. Data acquired from four permanent research plots (PRPs) located on the forestland and eight PRPs on the farmland were used. A number of tree- and stand-level models, stand structural indices, tree-rings, and climate characteristics were analysed to evaluate the hypotheses. The results show significantly higher means of DBH, tree height and basal area on the forestland compared to those on the farmland. There was a larger mean standing stem volume on the forestland (466 m^3^ ha^–1^) compared to farmland (770 m^3^ ha^–1^). Significant difference was observed between the mean DBH and mean stem volume of healthy trees compared to those of the trees with substantial damage (girth damage >1/3 of stem circumference). A greater extent of the girth damage was found on 86% trees on the farmland, while 54% damage on the forestland. About 62% bark-strip damage was further deteriorated by rot infection on the farmland, while on the forestland such an infection was only for 39% trees. The precipitation significantly positively affected the radial growth of trees that were largely affected by ungulate damages on the farmland.

## Introduction

The global ecosystem has been increasingly influenced by the anthropic activities while humans have gradually reshaped the planet, changing the living condition and environment for other organisms [1–4]. The main reason for such changes concerning the landscape formation and use is undoubtedly the population growth [4,5]. A deforestation in the past centuries was mainly due to expansion of farming [6,7] and decrease of a forested land area was due to cutting of forests for timber as building materials or firewood [8,9]. Changes in land use have also occurred in the last decades while the utilization of agricultural land has been much discussed elsewhere in the world, especially in the European countries [10–12].

However, the present situation in Europe is different from the past centuries as area of the forestland has gradually been increasing for the last 100 years [13]. As a consequence of the socio-demographic transformation of rural communities in the past, restoration of the abandoned agriculture land with afforestation and natural regeneration was carried out. For example, about 12.9 million hectares of the abandoned lands were afforested by European Union in 1995–2015 [12,14] and this resulted in an annual increase of afforestation by 0.4% since 1990 [15]. Mainly, the mountain areas in Europe were afforested due to de-intensification of agriculture or abandonment of the pasture or arable lands [16–20].

Situation in the Czech Republic is similar to the rest of other European countries, i.e., a large part of the non-forested land or abandoned agriculture land has been suitable for afforestation. The literatures show a vast area of abandoned agriculture land in this country was afforested in the past and another acreage is suitable for afforestation. For example Jarský and Pulkrab [21] reports 350,000 hectares of the unregistered, abandoned land that belong to the agricultural land resources, Podrázský and Štěpaník [22] reports 50,000–500,000 hectares of the land is suitable for afforestation. The fact is that total area of forestland in the Czech Republic has increased from 2.629 million hectares in 1990 to 2.667 million hectares in 2015 [14].

In the past, the abandoned agricultural land in Europe was often afforested by *Larix decidua* Mill., *Alnus glutinosa* /L./ Gaertn., *Pinus sylvestris* L. and *Fraxinus excelsior* L. [23–27]. However, *Picea abies* /L./ Karst. was often used for afforestation in the Czech Republic [28–30], mostly in the area of the Sudetes mountains [17]. Generally, *Picea abies* is one of the most widespread tree species in the North and Central Europe [31]. It has about 50.3% representation in the forests among all tree species in the Czech Republic [32]. About 86.1% forest cover on the study Orlické hory mountain is represented by *P*. *abies* [33]. *Picea abies* is used as one of the important tree species for afforestation in other European countries [25,34,35]. Many forest stands originated by natural regeneration after the abandonment of agricultural lands, especially deciduous tree species [26,36,37].

Forests established on the afforested agricultural land (first generation of forest) have specificities compared to standard forestlands. Those specialities may be fertile soil condition, faster tree growth, but poorer stability [35,38–40]. Fungal pathogen damages during a widespread root-rot disease across the forest stands, especially *P*. *abies* stands on the former agricultural lands are generally observed [41,42]. The questions would arise that what extent of stem rot and infection could be caused by soil condition and ungulate damages, especially by *Cervus elaphus* L. *Picea abies* is one of the most debarked tree species by ungulates, due to its thicker bark [43], especially in younger forest stands. All these facts will be enhanced by future climate change connected to the climatic extremes, increase of air temperature and lack of atmospheric precipitation [44–46]. Due to frequent drought and vitality loss by subsequent fungal and bark beetle attacks, large area of *P*. *abies* forest has been endangered where forest productivity has decreased and tree mortality has increased across Central Europe [47,48]. Moreover, the structure and diversity can play the important role in the growth response to the drought stress and attack of biotic pests [49,50].

At the beginning of the 21^st^ century, more than 20% harvested volume of the wood was damaged by rots in the Czech Republic [51], which was mainly due to the fungal decay by *Stereum sanguinolentum* (Alb. et Schwein.) on *P*. *abies*. The infected stands were mostly younger ones and they were already damaged by hoofed game [52–54]. An increased population of wild ungulates [55–57] has triggered the risk to the substantial damages of the stands by browsing and bark stripping [43,58]. By removing bark, the deer make the tree vulnerable to the fungal infestation, leading to the rotted wood [43,58,59]. The rot origination and spreading in relation to the production quality was investigated by several researchers, e.g., Vlad and Sidor [60] and Vlad [61]. The average stem volume loss due to fungal rot damages has been rising remarkably [62], and consequently this has increased the tremendous financial loss due to lower production quality [63,64].

The objective of this study was to evaluate the production (quantity and quality) and structural characteristics of the forest stands established through afforestation on the abandoned agricultural land (hereafter termed farmland) and *P*. *abies* forests originated by plantation (hereafter termed forestland). Specifically, this study aimed at determining (1) production and structural differences (in terms of diameter and height and stem volume of trees, and species diversity) between these two different stand types, (2) effects of climatic factors (precipitation, temperature) and bark stripping damages including wood rots on the radial growth and stem volume of *P*. *abies*, and (3) interaction between the loss caused by wild ungulates, production, diversity in each stand type. The study was intended to evaluate the direct effects of bark stripping on the production, structure, and indirectly to the economic and environmental impacts to the *P*. *abies* stands in terms of ongoing climate change condition. The basic hypothesis was that *P*. *abies* stands established on the abandoned agricultural lands would have a higher production potentiality; however, quality of the production would be lower as a result of the higher loss caused by ungulates and fungal rots. Another hypothesis was that structural differentiation would be identical for both the farmland and forestland.

## Materials and methods

### Study area

The study was conducted on the twelve permanent research plots (PRP) located on the southern part of the Orlické hory Mts (0.2–5.1 km from the Polish border), where *P*. *abies* is a dominant species. The study area also represents Protected Landscape Area in Natural Forest Area 25 in the Czech Republic. Specifically, all PRP belongs to Forest Management Rychnov nad Kněžnou and are lying between the villages Říčky v Orlických horách (WGS84 50°12'29"N, 16°27'11"E) and Neratov (WGS 84 50°12'52"N, 16°33'6"E). Four PRPs (PRP 1–4) are located on the forestland according to the forest management plan and other eight PRPs (PRP 5–12) were originated by afforestation of the abandoned agricultural land, and these stands were planted shortly after the World War II. The age of both the stand types is similar (67–68 years) according to the forest management plans. The elevation of the study area ranges from 620 to 700 m a.s.l. on the farmland, and from 720 to 740 m a.s.l. on the forestland. The bedrock is composed mostly of muscovite schists, and modal Cryptopodzols and Cambisols are prevailing soil types [23]. The slope ranges from 8 to 12 degrees with aspect facing towards the east.

The mean annual temperature on the study area is around 6°C and mean annual precipitation ranges from 900 to 1100 mm yr^–1^ with maximum in August (120 mm). The length of snow-cover season was 60 days with the average maximum snow depth between 30 and 40 cm. The length of the vegetation period (growing season) ranges from 120 to 130 days with mean precipitation 550 mm and mean temperature around 11°C [37]. The study area has a humid continental climate characterized by hot and humid summer and cold to severely cold winter (Cfb) [65] and it is one of the cold regions and CH7 subregions [66].

Vegetation cover on the studied PRPs ranged between 15–35% on the farmland and 25–65% on the forestland. Herb species (E1) in the farmland are represented by *Vaccinium myrtillus* (10%), *Calamagrostis villosa* (5%), *Avenella flexuosa* (5%), *Dryopteris dilatata* (+), *Oxalis acetosella* (+), *Maianthemum bifolium (+)*, *Luzula pilosa* (+) and *Rubus fruticosus* (+). Moss layer (E0) is formed by *Polytrichum formosum* (5%), *Polytrichum commune* (+), *Dicranum scoparium* (+) and *Dicranella heteromalla* (+). Similarly, on the forestland, *Vaccinium myrtillus* (20%), *Calamagrostis villosa* (15%), *Avenella flexuosa* (10%), *Dryopteris filix-mas* (+), *Oxalis acetosella* (+), *Trientalis europaea* (+) and *Homogyne alpine* (+) are found in herb layer (E1) and *Pleurozium schreberi* (5%) and *Polytrichum formosum* (+) are found in the moss layer (E0).

In terms of phytocoenology, all PRPs belong to *Piceeto-Fagetum mesotrophicum* (nutrition-medium spruce-beech sites) and alliance *Luzulo-Fagion sylvaticae* Lohmeyer et Tüxen in Tüxen 1954. Data on the bark stripping were extracted from the Forest management plans of the corresponding stand types (farmland and forestland), in which damages occurred during stand age of 20–30 years are reported. This is also mentioned by other researchers who have reported the most vulnerable age of *P*. *abies* stands to the game damages could be between 18–30 years [58,67]. All PRPs are situated within two hunting districts known as Neratov II with acreage of 1617 ha and Malá strana with acreage of 834 ha. Average number of the hunted game animals in the entire forest area (2451 ha) during 2016 and 2017 were about 58 individuals of *Cervus elaphus* L., 31 individuals of *Capreolus capreolus* L., 67 individuals of *Sus scrofa* L. and 18 individuals of *Ovis orientalis musimon* Pallas. Data of the hunted game species for whole Natural Forest Area Orlické hory Mts were made available since 1980 for the period when forest stands were significantly damaged and population of game increased. An increased number of their populations has been reported for different species, e.g., *Sus scrofa* (+780%), *Ovis orientalis musimon* (+45%) and *Capreolus capreolus* (+15%), however, population of *Cervus elaphus* has decreased (-32%) [68,69].

### Sampling and measurements

The Field-Map technology (IFER ‒ Monitoring and Mapping Solutions Ltd.) was used to establish twelve PRPs, each of them is 625 m^2^ (25×25 m or 20×31.25 m). The PRP dimensions were chosen according to shape of forest stand, each PRP was established in different stand group. The distance between PRPs ranges from 70 to 4120 m while mean distance is 490 m. The PRPs were located in the appropriate distance (40–90 m) from the meadows in order to reduce the plot edge effects [54,70,71]. The measured variables in the tree layer are: tree position, crown projection (at least in four directions perpendicular to each other), diameter at breast height (DBH ≥ 4 cm, accuracy 1 mm) by metal calliper, total tree height and height of the live crown base from ground level (accuracy 0.1 m) by hypsometer Haglof Vertex Laser II VL402 [72]. The damages caused by game animals were evaluated using the methodology of the Institute of Forest Ecosystem Research, Ltd. (IFER) adjusted with respect to the local conditions. The bark stripping was measured along the girth of the tree stem using a tape (to the nearest 1 mm) at breast height (130 cm), and subsequently divided into three categories: healthy trees (damaged girth ≤ 1/8 of stem girth), small damage (damaged girth > 1/8 and ≤ 1/3) and large damage (damaged girth >1/3). Bark stripping was quantified as the proportion of stem damaged (proportion of stem debarked) by large herbivores (e.g. *Cervus elaphus* L.) that peeled off the stem tissues of the tree external to the cambium with their teeth [59,73].

In 2016, increment cores of all *P*. *abies* trees on each PRP (332 individuals in total) were taken at DBH using a Pressler auger–perpendicularly to the stem axis downslope and upslope. Tree-ring widths with the occurrence of the rots were measured to the nearest 0.01 mm with an Olympus stereo microscope on the LINTAB measurement table (RINNTECH) and recorded by the TSAPWIN software (RESISTOGRAPH).

### Data analysis

#### Tree height, diameter and stem volume

We compared the differences in the individual tree characteristics such as tree height, diameter and stem volume from forestland and farmland using Wilcoxon rank-sum test in case of height and stem volume, Welch two sample t-test in case of diameter at breast height (DBH). We produced the height-DBH curves using the Näslund function fitted to our height-DBH data [*h* = *DBH*^2^(*a*+b*DBH)^−2^+1.3][74], where *h* stands for total tree height, *a* and *b* are parameters to be estimated. Only trees with DBH ≥ 4 cm) were included in the analysis.

#### Stand characteristics and diversity

For a general description of the stands, following characteristics were computed: tree density, basal area, stand volume [75], height-diameter ratio, periodic annual increment, mean annual increment, canopy closure, crown projection area, stand density index and supplementary species ratio.

To assess the stand diversity, following indices were computed using Sibyla 5 software [76]: Arten-profile index [77], diameter and height differentiation index [78], species diversity index [79], species evenness index [80], species richness index [81] and total diversity index [82].

The spatial distribution was evaluated using the index of non-randomness [83,84] and aggregation index [85] for all tree and understory layers. The criteria of diversity indices are shown in Table 1. The PointPro 2 software (CULS, Zahradník & Puš) was used for computation of the horizontal stand structure. The test of the deviation from expected values for a random point pattern was done by Monte Carlo simulation.

**Table 1:** Overview of indices describing biodiversity and their general interpretation.

Criterion	Quantifiers	Label	Reference	Evaluation
Horizontal structure	Index of non-randomness	*α* (P&Mi)	[83,84]	mean value *α* = 1; aggregation *α* > 1; regularity *α* < 1
	Aggregation index	*R* (C&Ei)	[85]	mean value *R* = 1; aggregation *R* < 1; regularity *R* > 1
Vertical diversity	Arten-profile index	*A* (Pi)	[77]	range 0–1; balanced vertical structure *A* < 0.3; selection forest *A* > 0.9
Structure differentiation	Diameter dif.	*TM*_*d*_ (Fi)	[78]	range 0–1; low *TM* < 0.3; very high differentiation *TM* > 0.7
	Height dif.	*TM*_*h*_ (Fi)		
Species diversity	Heterogeneity	*H´* (Si)	[79]	minimum *H´* = 0, higher *H´* = higher values
	Evenness	*E* (Pi)	[80]	range 0–1; minimum *E* = 0, maximum *E* = 1
	Richness	*D* (Mi)	[81]	minimum *D* = 0, higher *D* = higher values
Complex diversity	Stand diversity	*B* (J&Di)	[82]	monotonous structure *B* < 4; uneven structure *B* = 6–8; very diverse structure *B* > 9

#### Effect of girth damage on height, diameter and stand volume

Testing of differences in mean height and mean stem volume between the defined levels of stem girth damage (healthy tree, small damage, large damage) altogether with soil type (forest site, former agricultural land) was performed using the Kruskal-Wallis test. In case of DBH, testing of the difference was performed by analysis of variance (ANOVA). The multiple comparisons (Siegel & Castellan [86] in case of Kruskal-Wallis test, Tukey HSD test in case of ANOVA were used to determine the significant differences between variables of interest.

#### Tree diameter increment

Diameter increments of trees on the forestland and farmlands were evaluated via tree-ring analysis. Tree-ring increment series were individually cross-dated (removal of errors connected with the occurrence of missing tree rings) using the t-tests in the PAST4 software [87] and, subsequently, visually checked according to the Yamaguchi [88] method. The curves were detrended in a standard way and mean tree ring series were created in the ARSTAN software using the 30-year smoothing spline [89]). Analysis of the negative pointer years was done using the methods suggested by Schweingruber [90]. For each tree, the pointer year was tested as an extremely narrow tree-ring that does not reach 40% of the average increments of four preceding years. We considered the negative year when there was a significant increment reduction at least on 20% trees per PRP. For paired samples, the Wilcoxon rank-sum test was used for testing the differences between detrended tree-ring width index separately for healthy trees and trees with large damage (each observation is a difference in the detrended tree ring width index for one year between 1965–2016). For modelling diameter increment in relation to the climate characteristics (monthly precipitation and temperature) the DendroClim software was used [91]. Climate time series data were obtained from the meteorological station at Deštné in the Orlické hory Mts. (656 m a.s.l.; WGS84 50°18′24″N, 16°21′07″E). Time period 1965–2016 (after starting/affecting trees by bark-stripping) were used for dendrochronology analyses.

#### Relationship between bark stripping and stem rot

The relationship between number of trees damaged by bark stripping and stem rot was evaluated using the Fisher’s exact test.

#### Modelling stem volume

The generalized linear model (GLM) with gamma distribution was used for modelling tree stem volume based on DBH, girth damage and site type. The inverse value was selected as a link function (default setting in “glm” function in R [92] software). The following form of a linear model was used to fit data (R software notation):

Stemvolume=DBH+Relativecircumferencedamage+Sitetype Eq (1)

Selection of the independent variables included in this model was done from its easier application point of view whereby these variables can be easily measured. The model outputs from farmland and forestland were compared using various graphs.

#### Stand structure, productivity and game damage interactions

The interactions between structure, productivity and ungulate damage in relation to the effects of climatic factors were evaluated using the principal component analysis (PCA) on a centred and standardized dataset. The results of PCA were visualized in the form of an ordination diagram.

#### Software used

We carried out the data analyses using Statistica 12 (StatSoft, Tulsa) and R software [92]. Three-dimensional plots were prepared using Gnuplot 5.2. The PCA was accrued out using CANOCO 5 program (Microcomputer power).

## Results

### Tree height, diameter and stem volume

The mean values were significantly higher for tree height (Wilcoxon rank-sum test, W = 4776.5, p<0.001), DBH (Welch two sample t-test, t = 6.79, p<0.001), stem volume (Wilcoxon rank-sum test, W = 6859, p<0.001) on the farmland compared to the forestland (Table 2).

**Table 2:** Stand characteristics of the tree layer on permanent research plots 1–12 in 2016.

PRP		dbh	h	v	N	G	V	HDR	PAI	MAI	CC	CPA	SDI
	y	cm	m	m^3^	trees ha^-1^	m^2^ ha^–1^	m^3^ ha^–1^		m^3^ ha^–1^ y^-1^	m^3^ ha^–1^ y^-1^	%	ha	
1	67	30.6	21.52	0.68	720	52.9	487	70.3	9.5	7.27	86.3	1.99	0.82
2	67	33.5	22.15	0.81	496	43.7	403	66.1	7.0	6.01	79.6	1.59	0.66
3	67	34.2	22.8	0.86	400	36.8	346	66.7	6.5	5.16	83.3	1.79	0.54
4	67	35.6	23.36	0.98	640	63.8	627	65.6	8.5	9.36	87.5	2.08	0.93
5	66	42.9	30.68	1.80	512	74.1	920	71.5	9.5	13.94	86.7	2.01	0.99
6	66	39.7	31.65	1.53	512	63.2	783	79.7	7.0	11.86	79.7	1.60	0.97
7	66	47.9	31.28	2.26	304	54.7	688	65.3	7.0	10.42	78.9	1.56	0.72
8	66	42.4	27.19	1.67	304	42.6	508	64.1	6.0	7.70	72.3	1.28	0.60
9	66	41.3	30.26	1.67	480	64.2	814	73.3	8.0	12.33	76.9	1.46	0.88
10	66	42.6	31.56	1.84	448	63.7	826	74.1	8.5	12.52	76.6	1.45	0.86
11	66	36.1	26.66	1.20	704	71.8	843	73.9	7.5	12.77	86.0	1.97	0.98
12	66	37.4	27.97	1.31	592	64.8	777	74.8	8.5	11.77	81.8	1.7	0.92

*t* average stand age, *dbh* mean breast height diameter, *h* mean height, *v* average tree volume, *N* number of trees per ha, *G* basal area, *V* stand volume, *HDR* height-diameter ratio (slenderness ratio), *PAI* periodic annual increment, *MAI* mean annual increment, *CC* canopy closure, *CPA* crown projection area, *SDI* stand density index

### Stand characteristics and diversity

The number of trees on PRPs ranged between 304 and 720 trees ha^–1^, both on the farmland and forestland. Stand density index also varied on both stand types, and tree density on the farmland was lower by 14% compared to forestland, while larger differences were observed in the timber production. Total stand volume on the forestland was 466 m^3^ ha^–1^ (±122 SD), and 770 m^3^ ha^–1^ (±125 SD) on the farmland. *Picea abies* was a dominant tree species (90.8%) on all PRPs. Among other admixed tree species are *Larix decidua* Mill. (6.5%) and other tree species such as *Betula pendula* Roth, *Fagus sylvatica* L. and *Sorbus aucuparia* L. that account for less than 2% species composition. The mean annual increment of the stands fluctuated in the range of 5.16 (forestland PRP 3)– 13.94 (farmland PRP 5) m^3^ ha^–1^ yr^–1^, while the mean annual increment on the farmland was higher (by 67%) than on the forestland. The crown closure was in the range of 72.3–87.5%, which also showed a higher variability on the farmland, like canopy projection area.

**Fig 1:**
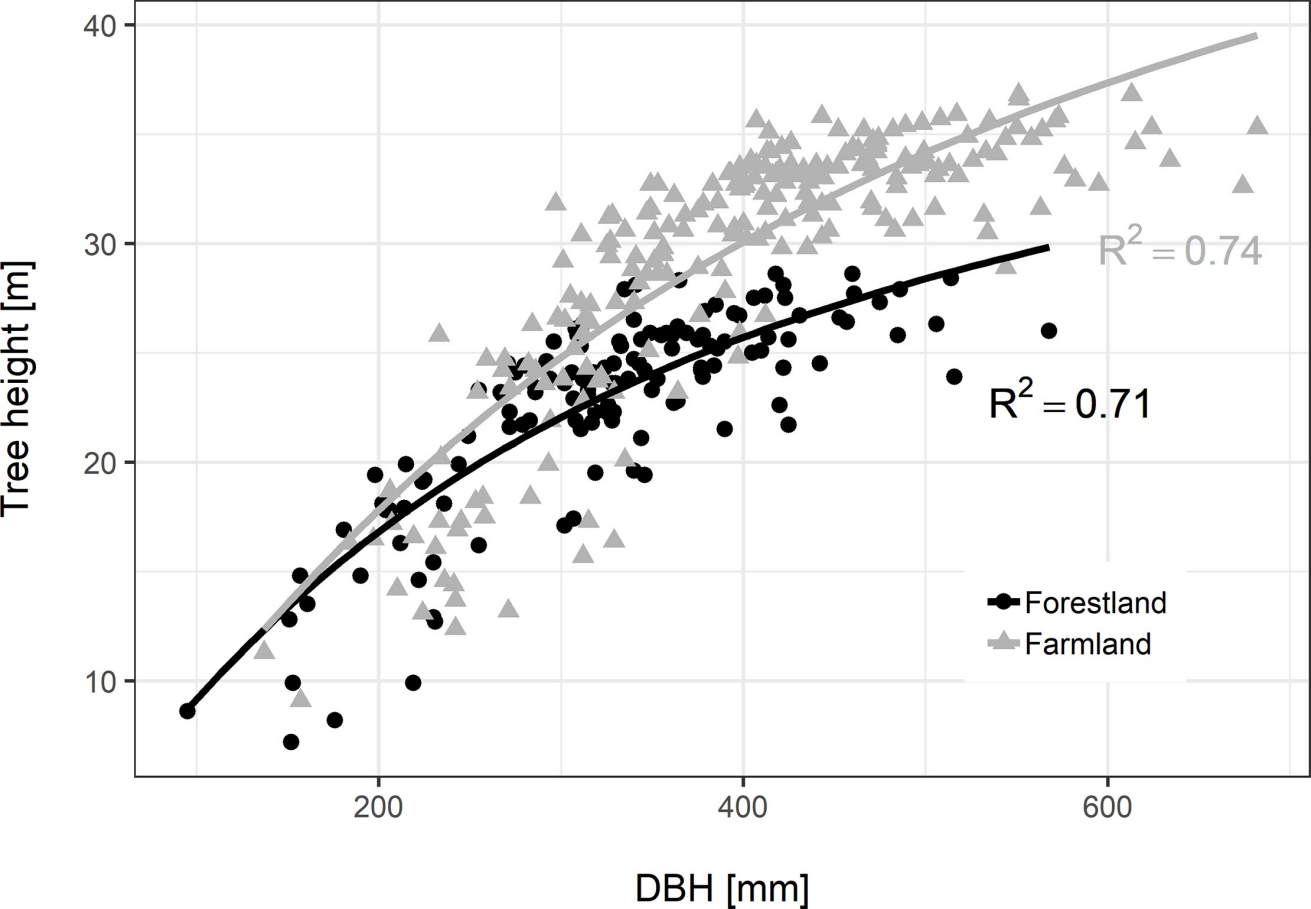
Relationship between diameter at breast height (DBH) and tree height of *Picea abies* on the forestland (PRPs 1–4; parameter estimates: *a* = 20.5, *b =* 0.15) and farmland (PRPs 5–12; parameter estimates: *a =* 23.8, *b =* 0.13).

The fitted Näslund’s function (Fig 1) showed the higher predicted heights with the same DBH on the farmland compared to those on the forestland. A proportion of height variations explained by this function for trees on the farmland was significantly higher (R^2^ = 0.74) than that on the forestland (R^2^ = 0.71). The predicted height difference in two stand types was about 11% for DBH = 30 cm, 20% for DBH = 40 cm and 27% for DBH = 60 cm.

Generally, horizontal structure was random for the forestland while spatial pattern for the farmland was mostly random (Table 3). However, PRP 6 and 12 (α = 0.05) were the exceptions, where distribution of individuals in the tree layer (*R* and α indices) was aggregated well. The *A* index indicated a slightly to strongly diversified vertical structure (*A* = 0.249–0.606) without significant difference between both variants. Diameter differentiation was at a low level (*TM*_*d*_ = 0.162–0.295), but height differentiation reached low to medium level (*TM*_*h*_ = 0.095–0.313) with a higher variability on the farmland. Species richness ranged from none or low to medium diversity (*D* = 0.000–0.350). Species heterogeneity was only at a low level (*H´* = 0.000–0.298) with a higher diversity on the farmland. Species evenness was low on the forestland (*E* = 0.000–0.226), while evenness on the farmland ranged from none or low to very high (*E* = 0.000–0.690). Total stand diversity, *B* suggested a remarkable homogenous structure (*B* = 2.776–4.739), except for a more diverse structure on the forestland PRP 1 (*B* = 5.489). Generally, more complex species diversity was observed on the forestland (B = 4.756) compared to the farmland (B = 3.919). For overview on all computed diversity indices, see Table 3.

**Table 3:** Diversity indices in the tree layer on the permanent research plots (PRPs) 1–12 in 2016.

PRP	α (P&Mi)	R (C&Ei)	A (Pri)	TMd (Fi)	TMh (Fi)	H´ (Si)	E (Pii)	D (Mi)	B (J&Di)
1	0.964	1.108	0.387	0.295	0.189	0.108	0.226	0.304	5.489
2	1.105	0.937	0.372	0.213	0.181	0.064	0.213	0.161	4.647
3	1.308	1.179	0.347	0.208	0.176	0.051	0.169	0.167	4.738
4	0.890	1.233	0.500	0.234	0.161	0.000	0.000	0.000	4.148
5	0.824	1.248	0.473	0.240	0.170	0.124	0.412	0.160	3.708
6	0.715*	1.324*	0.606	0.280	0.152	0.298	0.990	0.160	4.097
7	1.105	1.305	0.329	0.208	0.142	0.154	0.323	0.350	4.470
8	0.735	1.234	0.464	0.219	0.168	0.144	0.478	0.175	4.629
9	0.816	1.273	0.249	0.232	0.131	0.014	0.047	0.162	4.103
10	0.945	1.277*	0.291	0.162	0.095	0.000	0.000	0.000	2.776
11	0.823	1.124	0.408	0.280	0.313	0.031	0.103	0.153	4.247
12	0.702 *	1.315 *	0.605	0.200	0.231	0.000	0.000	0.000	3.318

*α* index of non-randomness, *R* aggregation index, *CS* index of cluster size, *A* Arten-profile index, *TMd* diameter differentiation index, *TMh* height differentiation index, *H´* index of species heterogeneity, *E* index of species evenness, *D* index of species richness, *B* stand diversity index;

*statistically significant (α = 0.05) for horizontal structure (*α* and *R*)

### Effect of girth damage on height, diameter and stand volume

**Fig 2:**
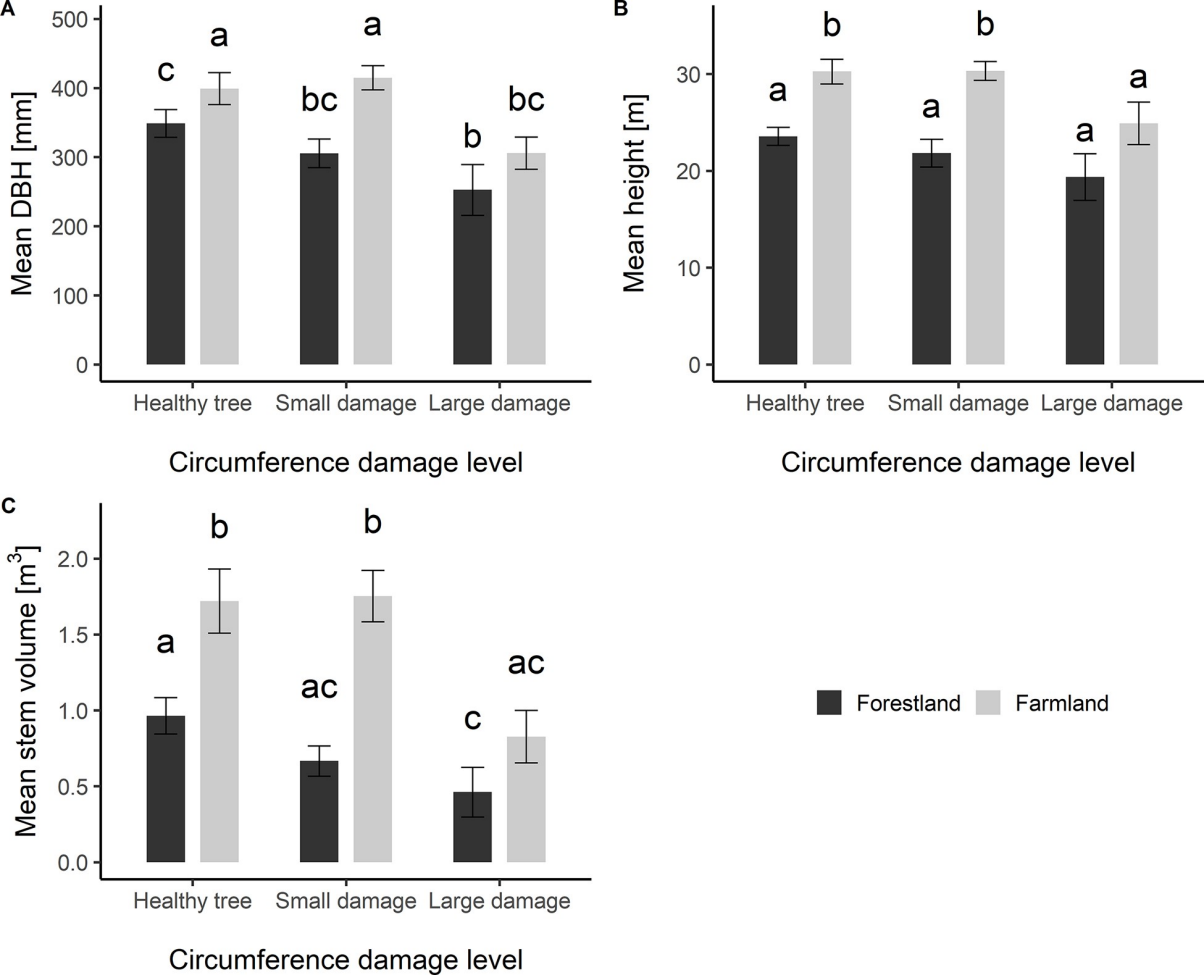
Statistical tests of differences on the mean height, mean DBH, and mean stem volume between the selected level of stem girth damages on the forestland and farmland. Different letters above the bars indicate significantly different variants. Error bars depict 95% confidence interval. Number of observations for each variant is depicted in the white box inside of a corresponding bar.

Differences in the mean DBH, mean height and mean stem volume were tested between different damage levels for both stand types. The Kruskal-Wallis test showed significant differences on the mean heights and mean stem volumes of trees for two stand types (p<0.001: chi-square test) and ANOVA test showed a significant difference of mean DBH (p<0.001: F-test). The differences on these three characteristics for both stand types are depicted in Fig 2.

In all cases, significantly different values were observed for healthy trees and trees with smaller girth damage in both stand types. For trees with larger girth damage, no significant difference was observed, however, the values for the farmland were higher in all cases.

Healthy trees on the farmland had a higher mean DBH by 14% compared to that on the forestland. For trees with small damages, this difference was 36%, but it was 21% on the heavily damaged trees. A similar pattern of the difference was observed for mean height. The differences were 28% for trees with no damage (healthy trees) on the farmland, 39% for trees with small damage and 29% for trees with large damage. There was a greater difference for mean stem volume. About 80% increase was observed for healthy trees and heavily damaged trees on the farmland, even a higher increase (160%) for trees with small damage.

### Tree diameter increment

**Fig 3:**
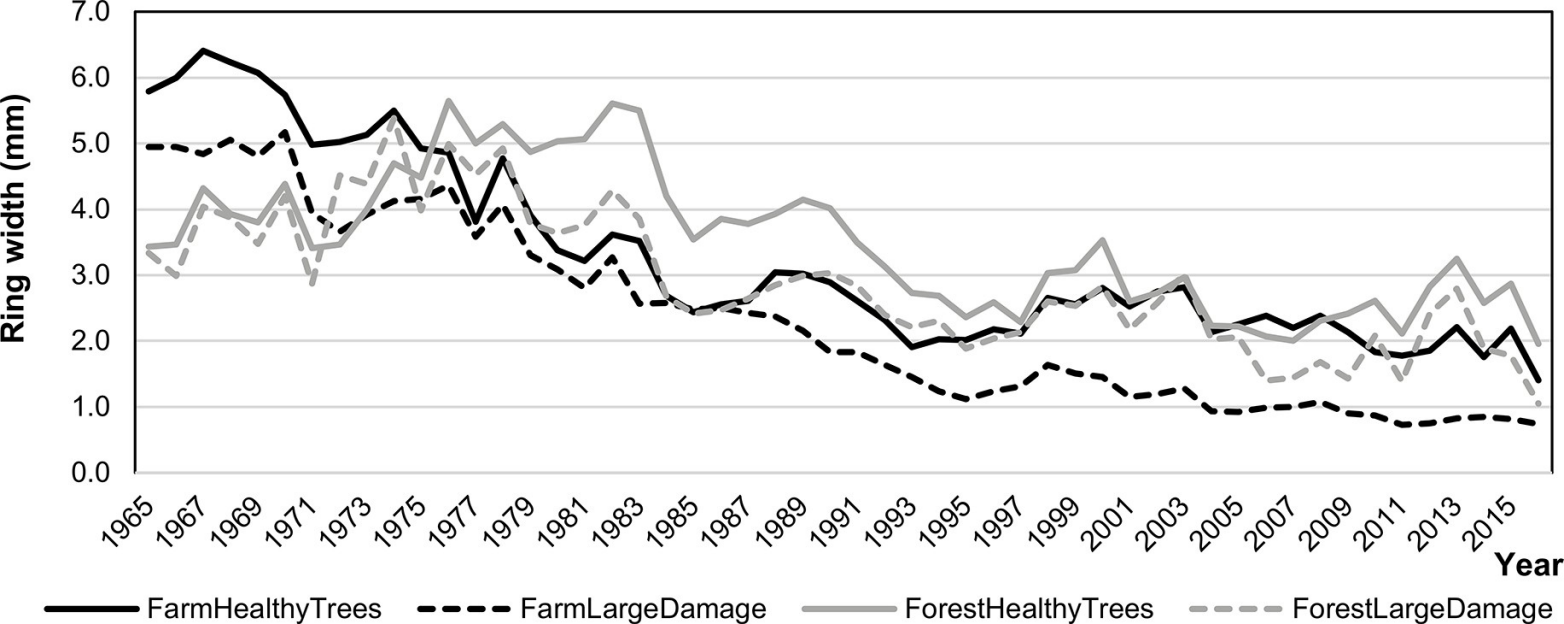
Average tree-ring width series on the forestland (grey) and farmland (black) by ungulate damages. Curves were produced only for healthy trees and trees with large damage as an illustration to show the effects caused by damages.

Tree diameter increments were analysed using the tree-ring width measurements on the collected core samples. The mean tree-ring width curves showed significantly high goodness-of-fits (t-tests ≥ 5.7). This consistency allowed to develop a local standard chronology for the *Picea abies* stands in the Orlické hory Mts. The regional standard tree-ring chronology indicates the relatively balanced radial growth in 1965–1982, interrupted by a decrease in 1983–1986. The year 1986 is a period of the increased radial growth, interrupted by a strong decrease in 1994, and then in 2001, 2004, 2011 and 2016. The radial growth of healthy trees on the farmland was higher until 1976 (stand age of 25 years). After that, the radial growth on the forestland was dominant, but this was negatively influenced by game damage on the farmland (Fig 3).

The years 1985, 1994, 2004 and 2016 were confirmed as the negative pointer years. There was a severe ice damage on the *P*. *abies* stands (historically the coldest January and February–average temperature -8.1°C, in 1965–2016 –average temperature -3.1°C) in 1985. Severe drought also occurred (the hottest and dry June and July; temperature > 16.7°C, precipitation < 104 mm, in 1965–2016 –mean temperature 14.4°C and 243 mm of precipitation) in 1994 and 2004, and there was a very dry summer (historically the driest vegetation season < 300 mm of precipitation, in 1965–2016 –mean temperature 14.4°C and 617 mm of precipitation) in 2016.

The detrended ring-width indices of the heavily damaged and healthy trees were compared separately for forestland and farmland. These indices were lower for the heavily damaged trees on both stand types, however, difference was significant for farmland (p = 0.047: Wilcoxon rank-sum test), while a non-significant result was observed for forestland (p = 0.24).

**Fig 4:**
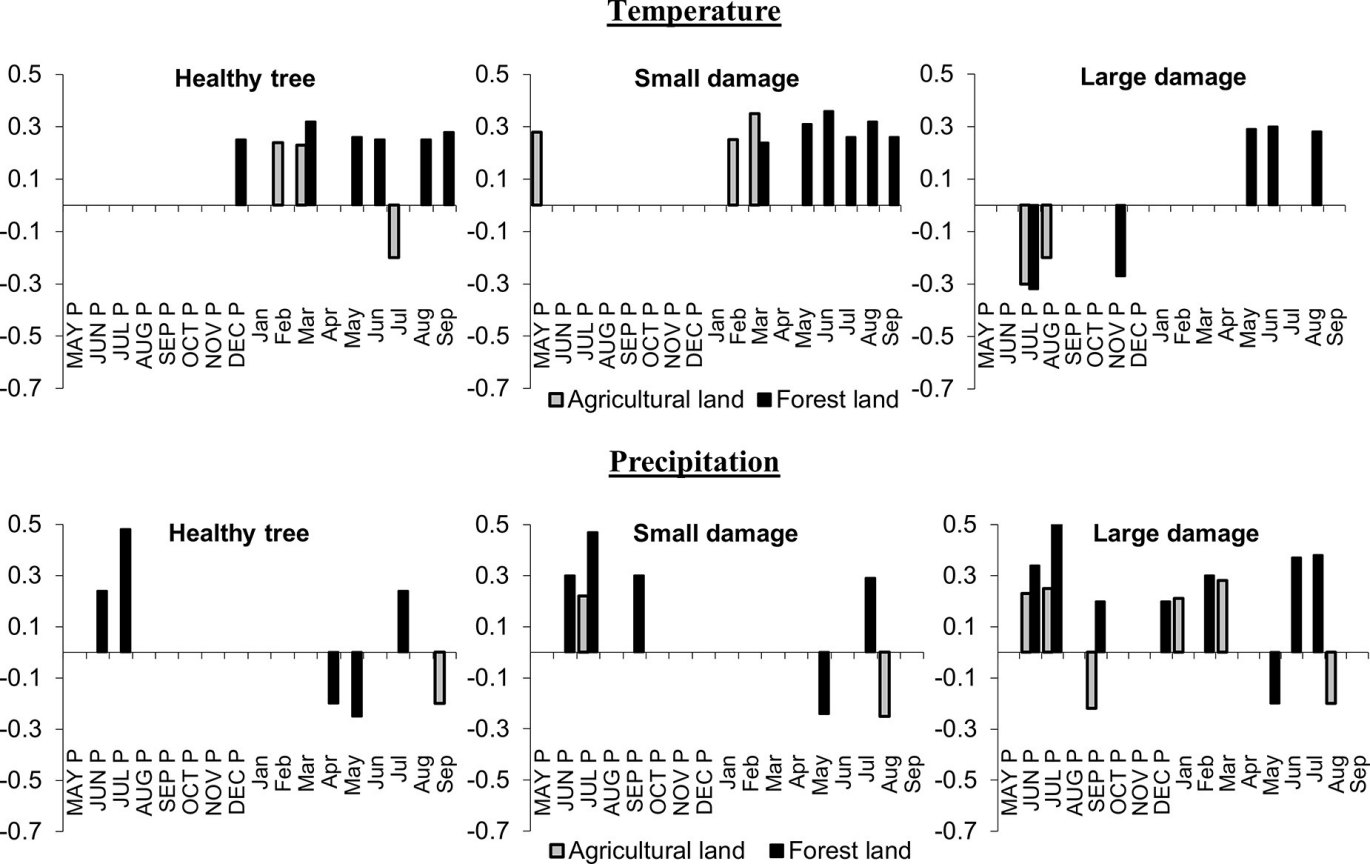
Correlation coefficients of tree-ring index chronology with monthly temperature (up) and precipitation (down) from May of the previous year to September of the current year for period 1965–2016 on the forestland (black) and farmland (grey) divided according to the damage caused by ungulates (bark stripping by *Cervus elaphus*); only statistically significant correlations are shown (α = 0.05).

Climatic factors for a period between 1965 and 2016 showed a significant effect on the diameter increments (Fig 4, Table 4). Temperature was identified as a major factor influencing the diameter increment of *P*. *abies*. Monthly and annual temperature had a higher effect on the radial growth of healthy trees (trees with no damage or small damage) compared to the effect of precipitation, while trees on the forestland were significantly more vulnerable to climatic factors. Temperature in the vegetation season had a positive effect on the radial growth of trees on the forestland, especially on June (r = 0.36). On the contrary, a more pronounced positive effect of temperature on the farmland was at the beginning of the current year, especially on March (r = 0.35).

**Table 4:** Pearson’s correlation coefficients calculated between *Picea abies* tree radial increment and climate for various combination of tree health status and climatic variables. Statistically significant correlations are in bold (p < 0.05) and underlined (p < 0.01).

***Variant***	***Temperature***					
	ActAnn	ActVeg	LasVeg	ActNon	ActI–III.	ActVI–VII.
Farmland Healthy trees	0.19	0.07	-0.05	**0.25**	**0.24**	0.06
Forestland Healthy trees	**0.28**	0.17	-0.09	**0.32**	**0.33**	0.07
Farmland Large damage	-0.13	**-0.29**	**-0.26**	0.05	0.08	**-0.31**
Forestland Large damage	0.00	-0.05	**-0.29**	0.05	0.14	-0.12
***Variant***	***Precipitation***					
	ActAnn	ActVeg	LastVeg	ActNon	ActI–III.	ActVI–VII.
Farmland Healthy trees	-0.05	**-0.26**	0.17	0.17	0.19	-0.10
Forestland Healthy trees	-0.08	-0.04	**0.32**	-0.05	-0.06	**0.24**
Farmland Large damage	0.17	-0.13	0.18	**0.36**	**0.33**	0.03
Forestland Large damage	0.16	0.19	**0.50**	0.05	0.05	**0.41**

ActAnn–annual value (mean temperature or sum of precipitation) of the given year, ActVeg–value in the vegetation season of the given year, LasVeg–value in the vegetation season of the previous year, ActVeg–value in the vegetation season of the given year, ActNon–value outside the vegetation season of the previous year, ActI–III–value in January–March of the given year, ActVI–VII–value in June-July of the given year.

Precipitation had a less effect on the diameter increment on the farmland compared to the forestland. A higher impact of precipitation on the radial growth was observed in the previous years with a maximum positive effect on July (r = 0.53) as in the current year (r = 0.38). A more pronounced positive effect of precipitation on the radial growth was observed for those trees, which were strongly affected by ungulates on the farmland. Generally, bark stripping and subsequent stem rotting were found more closely related to precipitation than temperature. For instance, on the farmland, only one pointer month was observed, while trees with severe damage were very vulnerable to draught (6 pointer months). For exact values of all computed correlation coefficients, see Table 4.

### Relationship between bark stripping and stem rot

Relationship between the numbers of trees damaged by bark stripping in relation to the stem rot is presented in Table 5. Significant differences were observed for both stand types (p<0.001).

**Table 5:** Numbers of trees by damage types for forestland and farmlands.

	**Forestland**		
		*Stem damage*	
		present	absent
*Stem rot*	present	33	2
	absent	51	70
	**farmland**		
		*Stem damage*	
		present	absent
*Stem rot*	present	104	3
	absent	65	25

**Fig 5:**
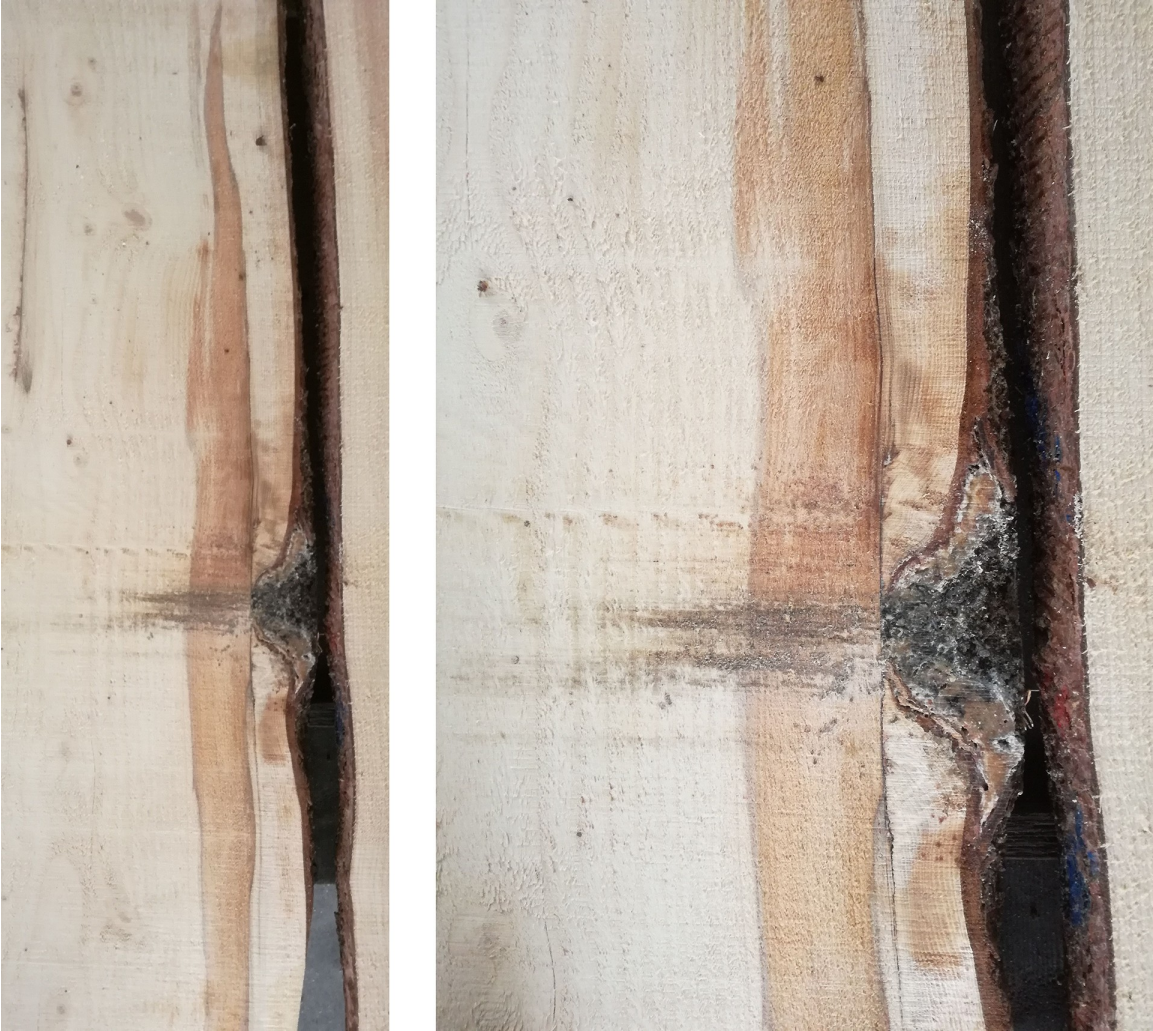
Red heart rot on *Picea abies* caused by *Stereum sanguinolentum* (Alb. et Schwein.) Fr. after preceding bark stripping by *Cervus elaphus* in the Orlické hory Mts.

Generally, only a few trees with stem rot but without bark damage were observed. On the farmland, most trees (104 trees) showed both damage types, and 59% trees damaged by bark stripping were also affected by subsequent rot infections. However, a different situation was found for forestland, where only 39% trees damaged by *Cervus elaphus* were also affected by rot infection. Most of the trees (70 trees) on the forestland were not damaged. The development of the rot infection caused by *Stereum sanguinolentum* in the *Picea abies* stem is illustrated in Fig 5. The beginning of the rot infection is located at the spot where the bark was stripped by *Cervus elaphus* and the infection has expanded vertically along the tree bole.

### Modelling stem volume

Results of the generalized linear stem volume model (GLM) (Eq 1) are presented in Table 6. The model includes effects of site type, relative girth damage and DBH on the stem volume variations within a particular stand type.

**Table 6:** Results of a generalized linear (GLM) model.

Variables:			
	Estimate	t-value	p-value
Intercept	2.09	24.1	**<0.001**
DBH	-0.002	-19.3	**<0.001**
Relative circumference damage	0.003	2.2	**0.032**
Site type	-0.41	-6.7	**<0.001**
	Value	DoF	
Null deviance:	165.23	270	
Residual deviance:	65.07	267	

**Fig 6:**
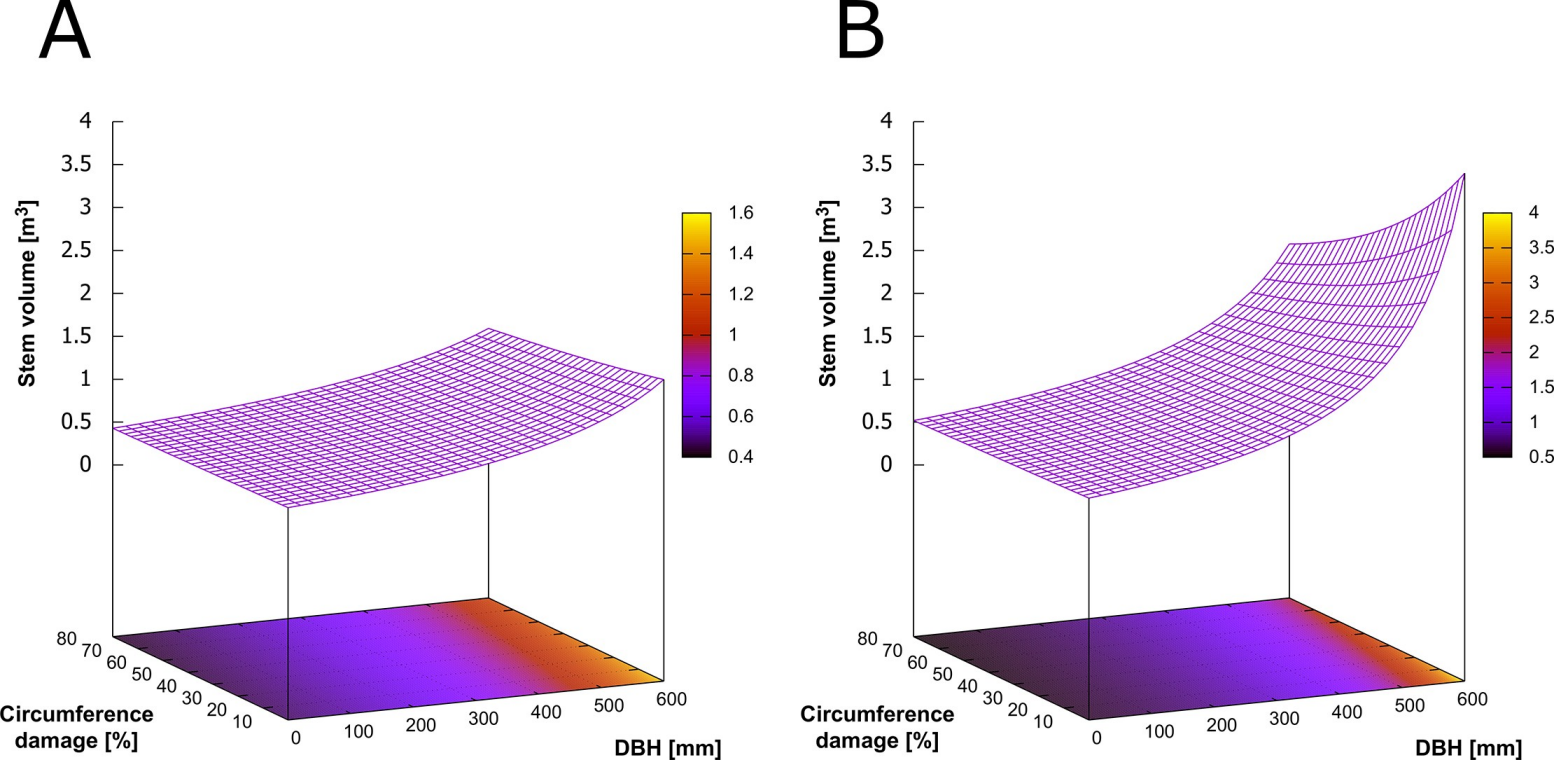
Graphical representation of the generalized linear model for the prediction of stem volume for forestland (A) and farmland (B).

The model (Eq 1) explained 61% of variation in the stem volume. All selected explanatory variables had significant effects on the stem volume. The stem volume was significantly affected by the percentage of damages on the girth (Fig 6). For instance, trees with DBH of 60 cm with 60% of stem girth damage reached only 58% stem volume of the healthy trees. With increasing DBH, this difference would be remarkably higher.

### Interactions of stand structure, productivity and game damage

**Fig 7:**
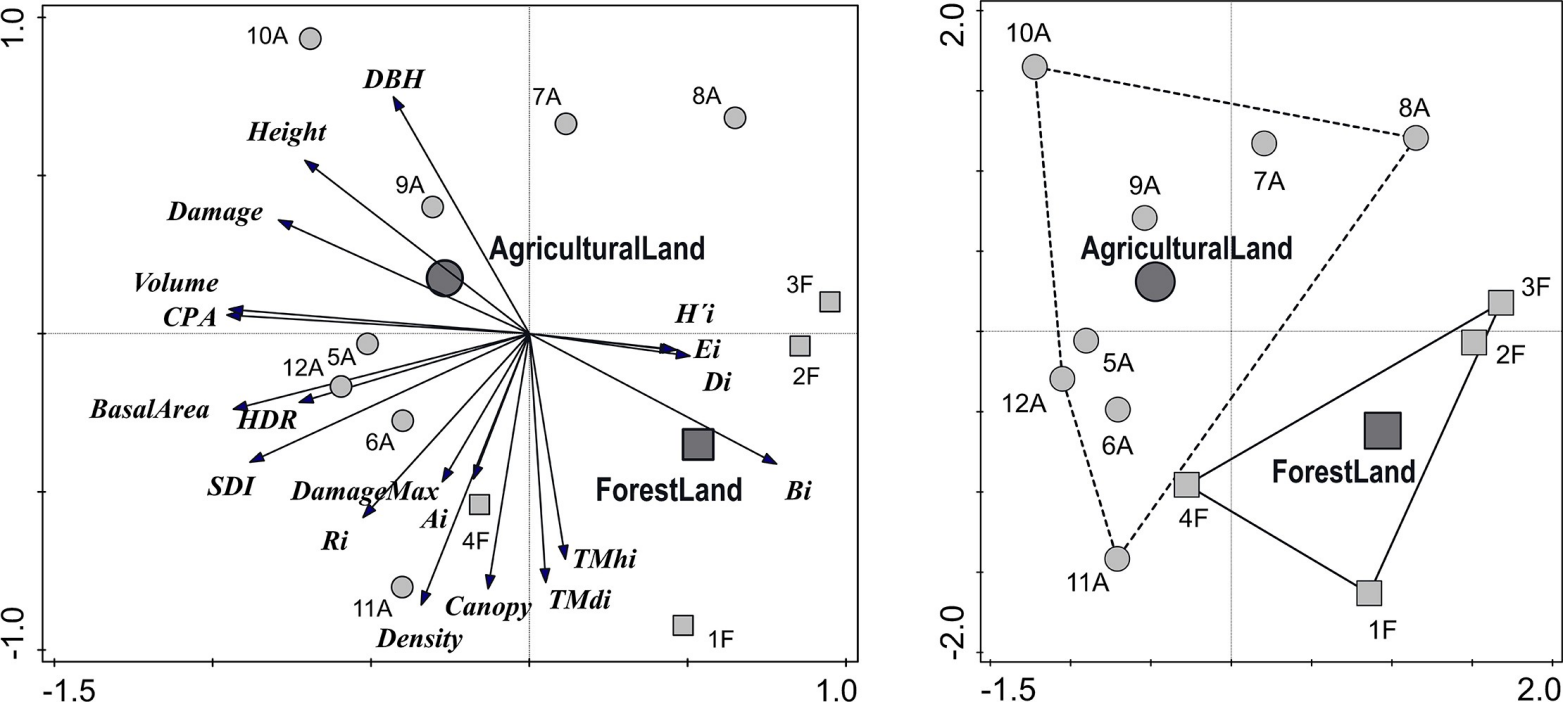
Ordination diagrams showing the results of PCA of relationships between stand characteristics (*Volume*, CPA *crown projection area*, *Basal area*, *DBH* diameter at breast height, *Height*, *Canopy* crown closure, *Density* number of trees, *HDR* height-diameter ratio, *SDI* stand density index/stocking), structural diversity (*Ai* Arten-profil index, *TMdi* diameter differentiation, *TMhi* diameter differentiation, *Ri* aggregation index), species diversity (*Ei* evenness, *Di* richness. *H´i* heterogeneity), complex diversity (B index), game damage (*Damage* percentage of damaged trees, *DamageMax* occurrence of damage >1/3 of stem), stem rot (*Rot*) and type of stand origin (● Foresland, ■ Farmland); Codes indicate the stand type and number of PRPs associated with it.

The PCA showed that the first ordination axis explained 37.5% variability (Fig 7). The first two axes explained 62.4% variability and all four axes explained 87.2% variability. The x-axis represents stand volume and crown projection area which were negatively correlated with tree species diversity. The second y-axis represents structural differentiation which was positively correlated with canopy closure while DBH showed negative correlation to both indices.

Overall stand diversity was positively correlated with species heterogeneity, richness and evenness, while these measures were negatively correlated with stand volume, height, diameter, ungulate damage, and stem rot. The stand types showed the lower mutual relationships between diversity indices compared to the differences in the production measures, especially in stand volume, increment, diameter and height. The PCA also show large differences among the individual PRPs. Differences within a particular stand type were remarkable, especially for PRPs on the farmland, where the marks of each record were relatively distant from each other whereas PRPs on the forestland showed a higher homogeneity. The stand type has no substantial effect on the occurrence of large girth damage or on the spatial patterns of trees.

## Discussion

The literatures [93–96] report that the *Picea abies* stands were established on the farmland in the past where adequate amounts of the nutrients were available. These stands are more productive compared to the stands on the forests with identical localities and stand conditions [39,40,97–99]. Moreover, higher timber production would be possible on the farmland, not only due to availability of more amounts of the nutrients, but also due to favorable physical properties of the topsoil (higher maximum capillary capacity and aeration of soil layers), more favorable soil sorption complex and microbial conditions [100–102]. Because of these more appropriate physicochemical properties of the soil and improved condition of light and temperature for initial growth stages of *P*. *abies*, the radial growth would be faster on the farmland compared to that on the forestland. Thus, the *P*. *abies* stands on the farmland would have a greater amount of assimilation apparatus, which is also connected to the positive effect of a greater proportion of the horizontal precipitation. Our findings are thus consistent with those published results. Analysis of the stem volume of large trees (bole up to 7 cm top diameter) shows that the production potential of *P*. *abies* stands on the farmland could be higher by 65% relative to that on the forestland. This also indicates the production potentiality of the farmland is higher (by 33%) than that listed in the growth tables [103]. Cukor et al. [40] has also reported this for the Orlické hory Mts where the *P*. *abies* stands are at the age of 60 years on the farmland had higher standing volume (by 19%) than on the forestland. Compared to the values given in the growth tables [96], standing volume is higher by 24–47%. Similar results were reported by Podrázský et al. [39] for the Bohemian-Moravian highlands, where *P*. *abies* stands on the farmland were 50 years and had higher standing volume (3%) than that on the forestland, and this value is higher by 50% compared to that in the growth tables [96]. The production potential of a particular stand type largely depends on the silvicultural tendings, which may have considerable influences on the stand growth (stand diameter and height) [104]. These studies also show that mean diameter in a particular tending of the *P*. *abies* stands at the age of 40 years increase with decreasing number of trees per hectare. It may be true for mean height also.

There were higher mean values of the variable of interest on the farmland compared to the forestland (DBH: 33.5 cm vs. 41.3 cm; height: 22.5 m vs. 29.7 m; basal area: 49.3 m^2^ ha^-1^ vs. 62.4 m^2^ ha^-1^). On the other hand, *P*. *abies* stands on farmland may also be vulnerable due to heavy rainfall, where severe water erosion can occur, especially after afforestation and on the steep slope [99,105]. During afforestation, it is necessary to consider applying the dewatering system through the deeper furrows on the contour lines for minimizing water erosion and make the water supply to the planted seedlings easier. It was also applicable to our cases, where artificial planting was carried out. In addition, the strips of pickled stones were established between the fields in the past, which were mostly covered by deciduous trees (especially with rowan and sycamore maple) in our study areas. Our results are comparable to those by Cukor et al. [40], which show that mean DBH of the *P*. *abies* stands at the age of 60 years on the farmland is higher (by 48–63%), mean height 11% higher, and mean annual volume increment 23–46% higher on the farmland than those given in the growth tables [96]. Podrázský et al. [39], on the other hand, concluded that high production of the *P*. *abies* stands on the farmland is not due to larger mean diameters, but due to more number of trees per hectare at about similar level of the mean heights.

Our results show that that horizontal structure of the tree layer is random for most of the PRPs, except 2 PRPs (6 and 12) of the farmland where structure is aggregated. Similar results were reported in previous studies for *Picea abies*–*Fagus sylvatica* stands, e.g., Vacek and Lepš [106], Zahradník et al. [107] in *P*. *abies* stands, Vacek et al. [108] and Králíček et al. [109]. For *P*. *abies* stands on the farmland, random to moderately regular structure was observed by Vacek et al. [99]. Horizontal structure of forest stands is largely influenced by ungulate damages [69] and silvicultural practices [107]. Deer are often clustering to the groups in forest [110,111], and this is associated with the aggregated distribution of the trees damaged by these animals [112]. Subsequently, heavily damaged trees are harvested, and an aggregated structure of tree layer can occur, as in our case on the farmland. In term of total biodiversity, results of PCA showed a higher complex diversity on the forestland compared to that on the farmland at an expense of the stand production characteristics.

A larger extent of the ungulate damages (damage of 85.8% trees on the farmland and 53.8% trees on the forestland) may be explained by extensive afforestation within a short period in 1950’s, which resulted in the even-aged *P*. *abies* stands in large area [17]. This may provide better shelter conditions for wild ungulates, where hunting was more difficult than in the uneven-aged stands. Many studies [52,53,113] show *P*. *abies* is one of the most vulnerable species to the bark stripping by ungulates. Other admixed tree species (larch, beech, birch, rowan) are also bark stripped, however, we did not carry out the study on them. Čermák and Jankovský [114] stated that *Cervus elaphus* might make the wound sizes ranging from few to hundredth cm^2^, for example, Čermák et al. [114,115] found wound size up to 1000 cm^2^. The wound size depends on the ungulate’s behaviour, tree properties, year, and season or weather [116,117]. Larger wounds mostly occur on the trees with larger boles [114,118,119]. Many studies [114,120,121] demonstrated that larger the wound and earlier the occurrence, higher would be the chance of wood being rotted in a larger extent with increasing bole size. Even small wounds can influence the tree for a longer time, for example, a wound of 5 cm width could be healed within 10–20 years, a wound of 5–10 cm width could be healed within 20–30 years, and wound larger than 10 cm width could be healed within 40 years [103,122]. Henžlík [123] reported that the quality of standing volume was diminished by 20–30% and this could be decreased by 10% due to bark stripping and browsing. The study of bark stripping on the Beskydy Mts. Showed, on an average, a loss of timber quality by 7–9% [114]. Simon and Kolář [124] reported a loss of the standing volume by 20–30% on the Jeseníky Mts. Study by Čermák and Malík from Prostějov Municipal Forests [125] showed that, on an average, 56% trees were bark stripped and browsed by *Cervus elaphus*, and out of this, 82% damaged trees were affected by red heart rot disease caused by *Stereum sanguinolentum* fungus. These studies also reported that trees with red heart rot were accounted for the highest proportion during age class III whereby 92% trees were damaged by bark stripping. The rot disease could affect 10–94% stem mass of trees, and disease might spread vertically with an average rate from 1.3 to 28.1 cm y^-1^. For example, rot disease could spread up to bole height from 1.25 m to 4.38 m, which also related tree age and development stage [63].

The extent of stem decay in the *P*. *abies* stands established on the farmland in the Neratov area has been assessed by Vacek (not published). In this locality, tree stems at 59 years of age (stand characteristics: average height 26 m, average diameter 38 cm and average volume 1.2 m^3^) were affected by rot within 1–9 m from the base of the stem. The average extent of decay measured from the base of the stem was 4.2 m. However, it is difficult to quantify losses caused by poorer quality of the wood. Economic losses were quantified in the Scandinavia, e.g. rots caused by *Heterobasidion* spp. were estimated to approximately €0.5–1 billion per year [126].

About 80% *P*. *abies* trees damaged by bark stripping and browsing by *Cervus elaphus* and subsequently affected by the rot caused by *Stereum sanguinolentum* in southern Russian taiga [127]. Similar damages are reported in the Polish part of Krkonoše Mts. [128]. About 8–28% loss in the value production of the *P*. *abies* stands due to bark stripping and browsing is reported by Eidmann [129] in the western part of Germany. The greatest damage of the *P*. *abies* stands made by *Cervus elaphus* was reported on 20–40 years of stand age [130], while Koltzenburg [131] and Čermák et al. [115] reported 15–30 years of age and Gill et al. [132] 18–38 years. In this range of age, tree stems have partly been self-pruned from the lower dead branches while bark is still smooth and suitable for stripping and browsing [114].

Among the ungulate species, *Cervus elaphus* accounts for *Picea abies* damage to a decisive extent. At around 1980 in the Czech Republic, 98% loss was incurred by bark striping and browsing that were caused by *Cervus elaphus*, in 1983 Fanta [133] reported 76% loss, followed by mouflon (21%), and the remaining 3% loss was due to *Cervus nippon* Temminck and *Dama dama* L. The damage caused by *Cervus elaphus* is a crucial problem for conifer stands in the Czech Republic [54]. A higher loss can be expected as a result of the increasing population density of the *Cervus elaphus*, not only on the local scale [134,135], but also on the national scale in the Czech Republic [136]. However, problems of the damages caused by *Cervus elaphus* are also reported to be solved in many other European countries [58,137,138].

The monetary value of the bark stripped stands is mostly from 70 to 95% of the expected value without incurred losses [129]. Similar value (73–92%) has been also reported by Šafránek et al. [64]. The comparison of different realization does not include the increment loss, it only reflects the loss of the produced timber supply. A reduction of the ungulate population should not be the only measure leading to a decrease in the damage caused by *Cervus elaphus*, but also positive results can be obtained by broader food supply [139].

The precipitation and temperature significantly influence on the radial growth of *P*. *abies* in relation to the damage caused by ungulates and stand types. On the Orlické hory Mts., lower temperature may be the limiting factor of the radial growth as on the foothills and mountainous areas of the Europe [109,140–142]. We found that trees growing on the forestland are more vulnerable to the effects of climatic factors compared to the *Picea abies* stands on the farmland due to the nutrient deficiency [99]. Trees with greater bark stripping and browsing damages and rotting would more suffer from higher precipitation deficit (or drought). In Switzerland, precipitation is reported to have significantly higher effect on the radial growth of the damaged trees compared to the healthy dominant or suppressed trees [140]. As in our case, tree-ring analysis of the damaged trees in Finland shows a strong positive correlation with June precipitation compared to that in the heathy trees [143]. The precipitation for healthy trees on the farmland had less effect on the radial growth, but might play key roles for the accelerated damages. As in Germany [144] and Norway [145], June and July temperature most significantly affected the radial growth of *P*. *abies* in our studied area also. Generally, healthy trees (or trees with no damage or a little damage) are characterized by the positive effect of temperature on the radial growth. However, lack of precipitation as a limiting factor is typical for growth of the severely damaged trees. The dendrochronological results together with other results presented in our article may be useful for making the effective harvesting plans of the *P*. *abies* stands in both forestland and farmland. The ongoing global warming with frequent occurrence of the extreme climate events, especially droughts [47,146,147] may be the greatest threat to damaged trees, which ultimately exert the negative influence on the stability and production capacity of the forest stands regardless of their origin.

## Conclusion

Our results suggested that there was a relatively low timber volume on the forestland (466 m^3^ ha^-1^) than that on the *Picea abies* stands established on the abandoned agricultural land (770 m^3^ ha^-1^). A higher extent of bark damages caused by *Cervus elaphus* was found on the farmland (bark stripping found on 85.8% trees) and that on the forestland (bark stripping found on 53.8% trees). Analysis of the radial growth indicated that trees with severe damages and decay significantly negatively affected by high precipitation deficit (drought), while healthy trees which were little affected by bark stripping and browsing were less affected by precipitation deficit. The trees with an impaired health status that was caused by bark stripping in the past might have been attacked by bark beetles to a greater extent in the last decade when global climate change might have already happened.

The damage caused by wild ungulates on the *P*. *abies* of stands of the Orlické hory Mts. is a much crucial issue both from economic and ecological perspective due to a greater extent of the degradation and decay of woods. Apparently, the most efficient method of alleviating losses of this type would be to reduce the population density of *Cervus elaphus* to a level that it could be compatible to the local environmental conditions and making the wintering enclosures in the forests. The presence of wolves has substantially contributed to alleviation of the losses caused by ungulates in the nearby Sudetské Mezihoří Mts. A reduction of the *Cervus elaphus* population remains a key factor that enhances the resistance of the *Picea abies* forests to other biotic and abiotic factors.

## Supporting information

S1 File Individual tree data. (XLSX)

S2 File Dendrochronological analyses data. (XLSX)
